# The Impact of Physical Activity at School on Eating Behaviour and Leisure Time of Early Adolescents

**DOI:** 10.3390/ijerph192416490

**Published:** 2022-12-08

**Authors:** Wojciech Kolanowski, Katarzyna Ługowska, Joanna Trafialek

**Affiliations:** 1 Faculty of Health Sciences, Medical University of Lublin, 20-400 Lublin, Poland; 2 Faculty of Medical and Health Sciences, Siedlce University, 08-110 Siedlce, Poland; 3 Institute of Human Nutrition Sciences, Warsaw University of Life Sciences, 02-787 Warsaw, Poland

**Keywords:** adolescence, eating behaviour, lifestyle, physical activity, school-aged children

## Abstract

(1) Background: Diet and physical activity (PA) significantly impact health. The aim of this study was to evaluate the impact of long-term increase in organized PA level at school on the eating behaviour and leisure time of early adolescences in the period from the age of 10 to 12. (2) Methods: Children born in 2007 (*n* = 278) in groups with a standard (control group) and increased level of organized PA at school (4 and 10 h a week) were subjected to an anonymous follow-up survey. The questionnaire included 22 questions related to the eating behaviour and ways and frequency of leisure time PA. The study was conducted in the same groups in three assessment sessions in September 2017, 2018 and 2019. (3) Results: During the course of the study, it was shown improvement of eating behaviour in the increased PA group whereas decline in the standard PA one. The share of children with good and very good scores of eating behaviour decreased in the standard PA group from 56.89% to 54.54% and increased in the increased PA from 58.06% to 60.29%. In the increased PA group children more frequently than standard PA ate vegetables, fruits, fish, poultry meat, wholemeal bakery products, milk, dairy products and drinking tea without sugar, as well as ate breakfast. The standard PA children more frequently than increased PA ate high sugar and/or fat content food as sweets, savoury snacks and drank fizzy drinks as well as fast foods. The frequency of often undertaken leisure time PA increased in the increased PA group from 67% to 74%, while decreased in the standard PA from 58% to 52%. (4) Conclusions: Increase in organized PA at school beneficially influenced early adolescents’ eating behaviour and frequency of leisure time PA during 2 years observation. It also increased early adolescents’ awareness of healthy diet. Increased PA at school helps shaping healthy lifestyle among early adolescents.

## 1. Introduction

More than one in three of school-aged children in the European countries are overweight or obese [1,2,3]. Unhealthy eating behaviour and low physical activity (PA) significantly increase the risk of obesity [4,5,6,7]. Global tendency to unhealthy eating during the transition from childhood to adolescence is observed [8,9,10,11,12]. Most adolescents often skip breakfast, eat low vegetables, fruit, fish, lean meat and dairy and excessive amounts of fatty and savoury snacks, as well as products and drinks with high sugar content [13,14]. Such eating behaviour increases the risk of obesity, diabetes, hypertension and other cardiovascular diseases, and cancer in adult life [15,16,17,18]. In particular, the diet of sport active adolescents should include not only the puberty needs, but also related to the increased PA [19,20,21,22,23].

The health benefits related to regular PA in children of school age are well documented [24,25]. These include improved development and function of the skeletal and circulatory systems, heart, lung and muscle efficiency, as well as physical fitness, and have a positive impact on body composition and weight [24,25,26,27]. However, in recent decades, an increase in sedentary behaviour among adolescents is observed [24,28]. The majority of school-aged children do not meet the recommendations related to PA, which has additionally increased during COVID-19 pandemic [29,30,31,32,33]. School should promote healthy lifestyles among children including organised forms of PA [33,34,35].

To reduce childhood obesity an increase in PA and healthy diet are needed [36,37,38]. The studies of Lipsky et al. [39] and Noll et al. [40] showed that in adolescents, favourable eating behaviour was associated with increased PA and better performance in sports. The studies of Całyniuk et al., (2014) [41] and Dortch et al., (2014) [42] showed that frequent PA resulted in better eating behaviour in school-aged children [41,42]. Nevertheless, some studies indicated that young athletes often had limited knowledge regarding a healthy diet [43,44,45,46]. Healthy eating and regular PA are the key to good health [47,48]. That is why the aim of this study was to evaluate the impact of a long-term increase in organized PA level at school on the eating behaviour and leisure time of early adolescents in the period from the age of 10 to 12. The hypothesis of this work assumed that an increase in PA at school by additional compulsory PE lessons positively affects the eating behaviour and leisure time PA frequency of early adolescents over time.

## 2. Materials and Methods

### 2.1. Subjects

This study was an observational follow-up cohort study with control. Eating behaviour and frequency of leisure time PA among early adolescents were assessed. The subjects were divided in two groups. The first group (control) consisted of children attending classes of standard level of organized PA at school (general education classes (GC)). The second group (experimental) consisted of children attending classes of increased PA at school (sports classes (SC)). Sport classes (SC) were not focus on a specific sport or discipline but on increased number of physical education (PE) lessons. The level of organized PA at school was reflected by the number of PE lessons, which was 4 h a week in the GC group and 10 h a week in SC. Six primary schools in Siedlce (an average size city in central Poland) participated in the study. In these schools, parallel classes with standard and increased number of PE lessons, 4 h for the GC and 10 h for the SC were organised. Children started their education in classes with an increased level of organized PA in the 4th grade of school [18,26,49]. PE lessons consisted of organised PA in the form of fitness exercises, sports forms of movement and team games.

The study was conducted in the same groups of children from September 2017 to October 2019. Children participating in the study were born in 2007, starting the 4th grade of primary school in 2017. At the start of the study, the children were ca. 10 years old, the study ended when children were ca. 12 years old and attended the 6th grade of school. The sample size was justified by a priori power analysis using the G*power software (Version 3.1.9.7; Universität Kiel, Germany) [50] with a type I error rate of 0.05 and 80% statistical power. The analysis indicated that 122 SC and 123 GC participants (total 245) were sufficient to observe significant large-sized acute effects (Cohen’s d = 0.80).

Adopted exclusion criteria: (1) incorrect age of the child (other than ca. 10 years of age at the start of the study), (2) not being promoted to the next grade of school, (3) lack of consent of parents or of the child for participation in the study; (4) a sick leave on the day of assessment, (5) diagnosed chronic conditions which could influence the results, (6) use of a special diet by the child (e.g., vegetarian, gluten-free, other) which could potentially influence the final result, (7) if the child was accepted to the SC or the GC during the study, as a result of a transfer from a different form or a different school, (8) incorrectly filled questionnaire. However, none of the parents of the children reported problems with diagnosed chronic diseases or use of a special diet.

### 2.2. Procedure

Children and their parents gave informed consent to participate in the study. The study was approved by the Ethics Committee at Siedlce University (No. 2/2016) and the positive opinion was expressed by the schools’ managements, parents and children. The parents gave their informed consent by filling the relevant form. All of them were informed about the purpose of the study and the confidential nature of the results.

The assessments were performed between 10:00 and 11:00 a.m. in the lesson rooms, in the presence of a teacher and the member of the research team. Before each assessment the children were asked if they wanted to participate. The assessment was preceded by a short instruction provided by the members of the research team, related to correct understanding of the questions, the children had the opportunity to ask questions [12]. The assessments were conducted in the same way in all schools. One month before each assessment day, the parents received a written note containing the message outlined above.

The study was conducted by a team of five licensed dietitians. The team members were trained in adopted methods, the confidentiality of results and coding. The team members knowingly and voluntarily agreed to participate in the study.

The children filled the same, anonymous questionnaire three times, in the beginning of the school years of the 4th, 5th and 6th grade of school. The initial assessment session was done between 1 and 30 September 2017, the second between 1 and 30 September 2018 and the third, and final between 1 and 30 September 2019. The results were systematically collected and analysed using Microsoft Excel 365 (Microsoft, Intentional Software, Washington, DC, USA) [51].

### 2.3. Questionnaire

The questionnaire was anonymous and included 22 closed questions with one answer to choose. The questions were related to eating behaviour (18 questions), self-assessment of eating habits and frequency of PA during leisure time (4 questions). The children were asked how often they consumed food products, such as meat (red and poultry), eggs, fish, milk, dairy products, regular and wholemeal bakery products and pasta, fine and coarse groats, rice, fruit, vegetables, fast foods, sweets, snacks and various drinks. Five possible answers were given: daily, every other day, once a week, once a month or never. In the section dedicated to leisure time PA, the questions were related to frequency and ways of spending leisure time, such as watching TV and using a computer or smart phone, reading, listening to music, sports games and plays, bike riding, walks, etc. The main study was preceded by a pilot one conducted among children at the approximate age of 10, from May to June 2017 in GC and SC, *n* = 43 (GC 22; SC 21). The pilot study was aimed to test of the procedure of questionnaire filling by the children [12].

The evaluation used a 5-degree rank scale: very good (rank 5), good (4), satisfactory (3), bad (2) and very bad (1). The obtained scores were added together and the average marks were determined. The high scores reflected the very good and good eating behaviour and leisure time PA level.

The following ranks were assigned to the frequency of eating foods recommended for healthy diet and to eating breakfast: 5—every day, 4—every other day, 3—once a week, 2—once a month, 1—never. A reverse classification was assumed, however, for food products which are not recommended in healthy diet. A modified classification was also accepted for white bread and pastry, red meat, as well as poultry, taking into account the recommendations for a healthy diet, i.e., the scale of every other day (rank 5), every day (4), once a week (3), once a month (2), and never (1). In the category of snacks eaten between meals, the highest scores were awarded to vegetables (5), fruit (4), followed by nuts (3), and the lowest scores were assigned to sweets, cookies (2) and savoury snacks (crisps, crunchy snacks–1 point). In the case of beverages, water received the highest score, followed by unsweetened fruit and vegetable juices and tea without sugar, while the lowest scores were assigned to fizzy drinks and sweet fruit juices. In the case of the questions related to lunch eaten at school, the highest scores were awarded for every day eating of lunch at school canteen (5), and home-prepared sandwiches (4), with much lower scores assigned to unsystematic lunching at school (3), buying food at the school store (2), and not eating at school at all (1).

The following ranks were assigned in order to determine the frequency of leisure time PA: 5 (very good: every day); 4 (good: on 4–6 days a week); 3 (satisfactory: on 2 or 3 days a week); 2 (low: on 1 day a week) and 1 (very low: no PA at all). The results were added together and the average scores were calculated. The averaged scores awarded for eating behaviour and leisure time PA for the entire period of the study were calculated on the basis of the average score scale for all questionnaires.

### 2.4. Statistical Analysis

Calculations were performed using the Microsoft Excel spreadsheet (Microsoft, Intentional Software, Washington, DC, USA) [51] and the Statistica 13 software (Stat Soft, Krakow, Poland) [52]. It was assumed that the statistical significance level α ≤ 0.05, i.e., *p* ≤ 0.05 indicated significant differences, while values *p* > 0.05 were interpreted as insignificant. The average values were calculated at the level of class profile, gender and age.

The following tests were done: the Shapiro–Wilk test assessing normality, the χ^2^-test (chi squared) and Cramer’s V test, the Mann–Whitney U-test and the Fisher–Snedecor test (F; single-factor variance analysis ANOVA). The ANOVA was used to study the impact of the group type (SC and GC) on the average score for eating behaviour and leisure time PA (rank 1–5). The above test was performed after the normality of the distribution and the variance uniformity were verified using the Shapiro–Wilk test. The Mann–Whitney U-test compared the criteria of assessment applicable to eating behaviour and leisure time activity between the initial and the final assessment in the GC and SC. It was also used to point out the differences between the groups size. The χ^2^ test was used in the analysis of eating behaviour and frequency of leisure time PA. The strength of the relationship between the groups profile and the eating behaviour (rank 1–5) and the frequency of leisure time PA was evaluated using the V Cramer (VC) test, which is based on the Pearson’ χ^2^ statistic and assumes inclusive values between 0 and 1, with the range assuming lack of a relationship at 0 and increasing to full relationship at 1.

Effect sizes (ES) for the obtained average scores for eating behaviour and for frequency of leisure time PA were calculated on the basis of the d Cohen value. The threshold values for the ES statistics were as follows: >0.2 low, >0.5 moderate, >0.8 high, >1.3 very high [53].

## 3. Results

### 3.1. Group Characteristics

During the first assessment session (4th grade of school, average age 10.27 years), a total of 291 questionnaires were collected (GC 151; SC 140). In the analysis 278 questionnaires were included (GC 143; SC 135; *p* = 0.780), 127 girls and 151 boys. Almost 6% of the GC questionnaires (*n* = 8) and 3.57% of the SC ones (*n* = 5) were excluded because they were not filled. During the second session (5th grade, age 11.27 years) 268 questionnaires were collected (GC 138; SC 130). In the analysis 261 questionnaires were included (GC 134; SC 127; *p* = 0.752), 125 girls and 136 boys. Almost 3% of the GC questionnaires (*n* = 4) and 2.5% of SC (*n* = 3) were excluded. During the third session (6th grade, age 12.27 years) 260 questionnaires were collected and the same number was included in the analysis (GC 133; SC 127; *p* = 0.420), 125 girls and 135 boys. A total of 819 questionnaires were collected throughout the entire study period (GC 410, SC 397). The final analysis included 799 correctly filled questionnaires (GC 410, SC 389), filled by 376 girls and 423 boys. In total 3% of the GC questionnaires (n = 12) and 2% SC (n = 8) were excluded from the final analysis.

### 3.2. Eating Behaviour

It was found that during the entire period of the study, on average, SC children more often than GC ones achieved very good scores (rank 5) and good scores (rank 4): 30.32% and 30.22% vs. 28.17% (F = 10.720; *p* = 0.025; ES = 0.520) and 28.90% (F = 0.985; *p* = 0.880; ES = 0.325), respectively (Table 1). Satisfactory score of eating behaviour (between good and bad–rank 3) were applicable to a similar share of children in both groups: 26.53% SC and 26.41% GC (F = 0.650; *p*= 1.205; ES = 0.185). Bad scores (rank 2) and very bad scores (rank 1) were achieved more often by the GC children (8.97% and 7.55%, respectively) than SC (8.63%; F = 0.879; *p* = 0.420; ES = 0.420) and 4.30% (F = 8.730; *p* = 0.031; ES = 0.052), respectively.

The SC children more frequently than GC declared eating of breakfast (*p* = 0.020), milk (*p* = 0.043), vegetables (*p* = 0.039), fruit (*p* = 0.041), wholemeal bakery products (*p* = 0.025), coarse groats (*p*= 0.028), poultry (*p* = 0.036), fish (*p* = 0.047) and drank tea without sugar (*p* = 0.047). The GC children more frequently than SC ate white bread and bakery products (*p* = 0.038), fine groats (*p* = 0.035), processed meats (*p* = 0.002), red meat (*p* = 0.014), as well as sweets and savoury snacks between the main meals (*p* = 0.040).

The average number of meals per day was similar in both groups during the entire study period. More than half of children declared eating of 4 meals per day (GC 50.67%; SC 52%; *p* = 0.810), 5 meals were declared by 32.16%, and 3 meals by 16.50%. None of the children ate 2 or 1 meal a day. The SC children were more likely than GC to eat breakfast (63.33% vs. 62%; *p* = 0.020). The eating of lunch was declared by 76% of SC children and 70.66% of GC children. Lunch was eaten at school canteens by 50% of children (52% SC; 49.33% GC; *p* = 0.058), and sandwiches brought from home by 22.66% of children (SC 24%; GC 21.33%; *p* = 0.888). The GC children were more likely to receive money to purchase lunch on their own than SC children (GC 16%; SC 13.33%; *p* = 0.031). The children indicated eating fruit as the most frequently declared snack between meals (GC 37%; SC 40%; *p* = 0.057). The GC children more frequently than SC snacked between meals sweets (26.33% vs. 22.33%) and savoury snacks (24% vs. 21%) (*p* = 0.040).

On average, 37% of children declared every day eating of milk and/or dairy products (GC 35.33%; SC 38.67%; *p* = 0.043). Approximately 21% of children in the GC group and 17% in the SC drank milk and ate dairy products only once a week (*p* = 0.007).

Fruit and vegetables were eaten once a day on average. However, the SC children ate fruit and vegetables more frequently than the GC. The every day eating rate of vegetables was 38.66% in the GC group and 46% in the SC (*p* = 0.039), and of fruit: 45% and 49%, respectively (*p* = 0.041). The children declared more frequent eating of fruit than vegetables, on average.

The frequency of white bread and bakery products eating was high. The average rate of every day eating (rank 4) was 62.50% (GC 64.68%; SC 60.33%; *p* = 0.038), every other day (rank 5) 25.33% (GC 25.33%; SC 25.33%) on average. Every day eating of wholemeal bakery products (rank 5) was 13% (GC 9%, SC 17%; *p* = 0.025), every other day 25.83% (GC 23.33%; SC 8.33%; *p* = 0.002). Despite the relatively infrequent consumption of wholemeal bread, it was significantly more frequently eaten by the SC children than GC (*p* = 0.025).

Coarse groats, oats and wholemeal pasta were rarely eaten, on average only 7.5% of children declared eating them every day (GC 6.67%; SC 8.33%; *p* = 0.028). The GC and SC children most frequently ate those products once a week (GC 54.33%; SC 54.33%). White rice, fine groats, standard pasta and flakes were typically eaten every other day (GC 32.00%; SC 23.67%; *p* = 0.055) and once a week (GC 49.34%; SC 58.00%; *p* = 0.351).

About half of the children ate eggs once a week (GC 56.33%; SC 55.00%; *p* = 0.794) and just under a quarter of them–every other day (GC 23.67%; SC 23.67%).

The GC children more likely than SC indicated every day eating of processed meat products (rank 4) than SC (28.00% vs. 23.33%; *p* = 0.002). Eating of processed meat products every other day (rank 5) was declared by 36.67% SC children and 32.00% GC (*p* = 0.037).

Eating of poultry meat was declared on average more frequently by the SC children than by the GC. On average, 43% of children ate poultry meat every other day (rank 5) (GC 42.00%; SC 44.00%; *p* = 0.036). Red meat was slightly more frequently eaten by the GC children than SC. Red meat was most frequently eaten every other day, with the average rate of 39.00% indications (GC 40.33%; SC 38.67%; *p* = 0.014).

Fish were eaten much more frequently by the SC children than GC. Fish was eaten once a week by average 49.66% of children (GC 46.00%; SC 53.33%; *p* = 0.020). Fish eating once a month was indicated by 28.33% of children (GC 26.34%; SC 30.33%; *p* = 0.002). Approximately 18.5% of children did not eat fish at all, which was more than twice often in the GC group than SC (GC 25.33%; SC 11.67%; *p* = 0.023).

The most frequently declared rate of fast food eating (average 65.83%) was once a month (GC 63.00%; SC 68.67%; *p* = 0.824). Fast food was never eaten by 6.33% of GC and 12.00% of SC children (*p* = 0.021).

Water was the most commonly chosen drink (rank 5) in both groups (GC 56.67%; SC 55.00%; *p* = 0.047). Tea without sugar was drunk by 17.00% of the SC children and 10.00% of GC (*p* = 0.037). Fizzy drinks were most frequently drunk by 9% of the children (11.67% GC; 6.33% SC; *p* = 0.047), and fruit juice by 16.15% (SC 17%; GC 15.33%; *p* = 0.065). On average, non-sweetened fruit and vegetable juices (rank 4) were drunk by only 5.5% of the children (SC 4.67%; GC 6.33%; *p* = 0.621).

#### 3.2.1. Eating Behaviour in Particular Sessions

It was shown that during the course of the study the average number of meals a day decreased from 5 to 4, which was especially noticeable in the GC group. The frequency of eating sweets and savoury snacks increased; milk and dairy products, vegetables, fruit and wholemeal bakery products also increased in both groups (Table 2, Table 3, and Table 4). Eating of coarse groats, wholemeal pasta and brown rice, as well as eggs, was the highest in the middle of the study period (average age 11.27 years). However, fine groats, white rice and standard pasta were eaten much more frequently than wholemeal. White bread was more frequently eaten in the initial session and decreased with age, both in GC and SC. The eating frequency of processed meat products, as well as red and poultry meat increased during the course of the study in both groups. The children more frequently chose poultry meat than red meat in the entire study period, both in the GC and SC group. A decrease in fish eating was observed, especially in the GC group. Fast food consumption was low, however it increased over time and the most frequent consumption was observed in the final session. The GC children more frequently ate fast food than SC, especially in the final assessment session. The children most frequently chose water and tea without sugar as their drinks. Fizzy drinks were drunk more frequently by the GC children than SC. On average, the SC children received higher ranks in eating behaviour than the GC.

The lowest level of good and very good ranks was noted in the initial session of the study (GC 56.89%; SC 58.06%). The highest share of very good and good ranks was noted in the middle of the study period (GC 60.28%; SC 63.31%; *p* = 0.034). The highest share of bad and very bad ranks was noted in the final session, more often in the GC group than SC one (GC 19.90%; SC 14.21%; *p* = 0.010).

#### 3.2.2. Changes in Eating Behaviour over Time

**Figure 1:**
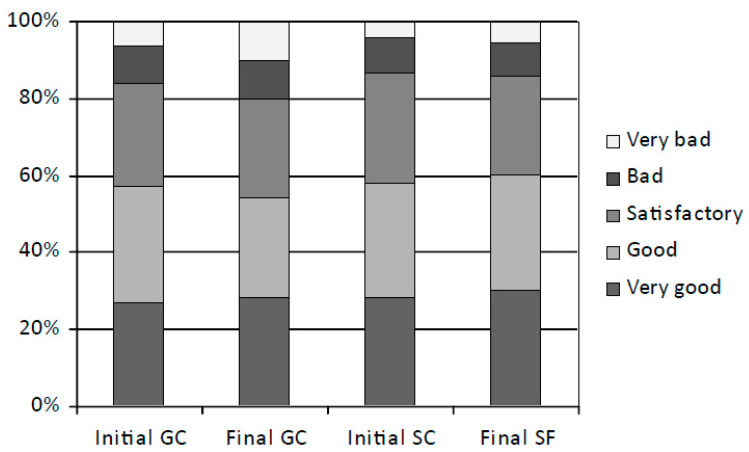
Comparison of eating behaviour between the initial (2017) and the final (2019) assessment sessions, %. GC—control group of standard PA at school (4 h a week); SC–increased PA at school (10 h a week).

The changes in eating behaviour over time were measured by the comparison of average results of the initial and final assessment sessions (average age of 10.27 and 12.27 years, respectively). In the final session, children achieved better results on average, which indicated more favourable eating behaviour than in the initial session (Figure 1). The percentage of children with very good score (rank 5) of eating behaviour increased in the GC group from 26.83% to 28.21% (*p* = 0.148) and in SC from 28.17% to 30.18% (*p* = 0.012). The percentage of children with good score of eating behaviour (rank 4) decreased significantly in the GC group from 30.06% to 26.33% (*p* = 0.015) and slightly increased in the SC from 29.89% to 30.11% (*p* = 0.062). The percentage of children with satisfactory score of eating behaviour (between good and bad–rank 3) decreased in the GC group from 27.28% to 25.56% (*p* = 0.040) as well as in the SC from 28.55% to 25.50% (*p* = 0.014). However, this decrease was replaced by an increase in better scores in the SC group and worse in the GC. The percentage of children with bad and very bad scores of eating behaviour significantly increased in the GC group from 15.83% to 19.90% (*p* = 0.003) and insignificantly in the SC from 13.39% to 14.21% (*p* = 0.780).

During the course of the study the most frequent score of eating behaviour was the satisfactory and very good–on average 29.56% (GC 28.90%; SC 30.22%; *p* = 0.048) and 29.24% (SC 30.32%; GC 28.17%; *p* = 0.025), respectively. Nearly 15% achieved the lowest scores, i.e., bad and very bad (GC 16.52%; SC 12.93%; *p* = 0.024). On average, the SC children achieved more very good scores than the GC. Between the initial and the final assessment sessions the share of children with good and very good scores of eating behaviour decreased in the GC group from 56.89% to 54.54% and increased in the SC from 58.06% to 60.29%. Finally, during the course of the study improvement of eating behaviour was shown in the SC group, whereas decline in the GC.

### 3.3. Frequency of Leisure Time PA

Not all children were adequately physically active during leisure time. It was observed that as children grew older, they tended to sedentary leisure time more frequently (e.g., watching TV, using phones, computer). During the initial stages of the study, 86% of the SC children and 75% GC (*p* = 0.012) preferred physically active ways of spending leisure time (playing, training sports) in most weekdays. However, a significant decrease in this percentage was observed in the final stage, especially in the GC group (SC 78%; GC 59%; *p* = 0.032).

Despite of frequency, on average children most often spent their leisure time involved in sports activity: 76.16% on average (SC 82.33%; GC 70.00%), while 23.83% preferred sedentary leisure time, such as watching TV (SC 17.67%; GC 30.00%; χ^2^ = 9.286, df = 1, *p* = 0.030, VC = 0.253).

The vast majority of children (SC 96.00%; GC 94.00%) were aware of the beneficial effects of a healthy diet on sports performance (χ^2^ = 0.286; df = 1; *p* = 0.596; VC = 0.053). During the initial stage of the study, 74% of the SC children and 53% of GC (*p* = 0.042) claimed to be eating a healthy diet, in the second session 64% and 82%, respectively (*p* = 0.008), and in the final assessment, 50% and 85% (*p* = 0.003). During the course of the study, the SC children with age more frequently indicated their diet was not healthy enough. However, a reverse trend was observed in the GC group. The SC children probably were more aware of healthy diet than GC ones. Overall, on average most children assessed their diet as healthy (GC 76.67% and SC 63.67%), while 38.66% as unhealthy (GC 23.33% and SC 36.63%; χ^2^ = 2.631, df = 1, *p* = 0.117; VC = 0.163).

The frequency of leisure time PA during the entire period of the study is presented in Table 5. On average leisure time PA was undertaken on 7 days a week by 32% of the children (GC 25%; SC 39%; F = 7.101; *p* = 0.012; ES = 0.052), and between 4 and 6 days–43.5% (GC 38%; SC 49%; F = 5.258; *p* = 0.050; ES = 0.032). Leisure time PA undertaken on 2–3 days a week was most frequently indicated at the age of 10.27 years (the initial session) (GC 33%; SC 33%; F = 0.002; *p* = 2.982; ES = 0.625). It was observed that the frequency of leisure time PA increased with age in the SC group, whereas decreased in GC.

On average nearly 40% of children undertook leisure time PA on 4–6 days a week and 30% declared it every day. The average percentage of very good scores (rank 5: every day PA) throughout the entire period of the study was higher in the SC group than CG (34.67% and 25%, respectively; F = 12.710; *p* = 0.021; ES = 0.62). A good score (rank 4) was noted in 32.67% of the GC group and 41.67% SC (F = 9.240; *p* = 0.048; ES = 0.320). A satisfactory score was achieved by 21.33% of the SC children and 33.67% of GC (F = 4.180; *p* = 0.023; ES = 0.41). Bad score (rank 2) and very bad (rank 1) were noted more frequently in the GC group than SC. On average 2.33% of the SC children and 4% GC reported leisure time PA on one day a week (rank 2; F = 3.320; *p* = 0.480; ES = 0.320). Almost 5% of the GC children very rarely or did not undertake leisure time PA at all (rank 1), while in the SC group no one did obtain rank 1 (F = 12.620; *p* = 0.002; ES = 0.62).

**Figure 2:**
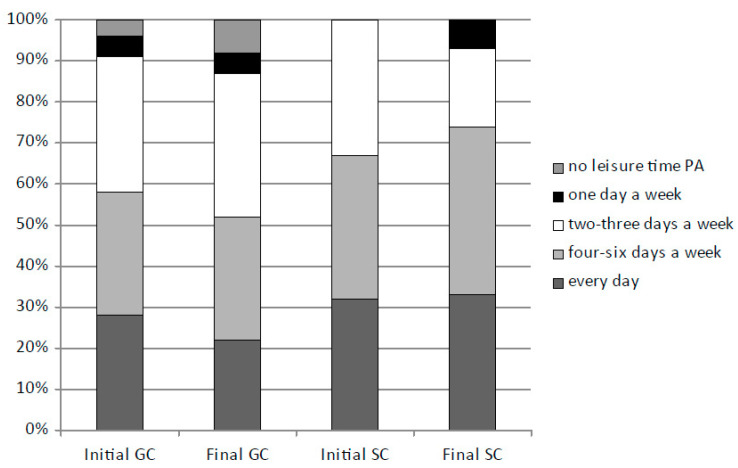
Comparison of frequency of leisure time PA between the initial (2017) and the final (2019) assessment sessions, %. GC—control group of standard PA at school (4 h a week); SC—increased PA at school (10 h a week).

Leisure time PA was undertaken on 2–3 days a week (rank 3) by 33.67% of the GC children and 21.33% SC (*p* = 0.003). Every day leisure time PA was undertaken by 34.67% of the SC children and 25% GC (χ^2^ = 12.612, df = 4, *p* = 0.013; VC = 0.357) (Figure 2). This decreased in the GC group from the initial value of 28% (age 10.27 years) to 22% (*p* = 0.014) in the final session (age 12.27 years). In the SC group leisure time PA was undertaken by nearly 35% on average, with a slight upwards trend. It was undertaken on 4–6 days a week by 37.17% of the children on average (GC 32.67%; SC 41.67%; *p* = 0.003), and one day a week by 3% (GC 4%; SC 2.33%; *p* = 0.048). The SC children more frequently undertaken leisure time PA than GC. In the GC group, the percentage of children undertaking leisure time PA very rarely or not at all increased from 4% (initial session) to 8% (final session). However, the SC children were somehow leisure time physically active during throughout the entire period of the study, and in the final session only 7% were active only on 1–2 days a week. In the SC group, the percentage of children undertaking leisure time PA only on 2–3 times a week decreased (from the initial 33% to 19%; *p* = 0.005), while the percentage of children leisure time physically active on 4–6 days a week increased (from 35% to 41%; *p* = 0.031). Finally, it was shown that the frequent leisure time PA (every day and 4–6 times a week) during the 2 years of the study increased in the SC group (from 67% to 74%) and decreased in GC (from 58% to 52%).

## 4. Discussion

Unhealthy diet and inadequate PA are the main risk factors of obesity and related diseases in school-age children [1,2,54]. Many authors indicated a positive relationship between healthy diet and PA level [39,55,56,57]. The study of Lipski et al. (2017) and Noll et al. (2019) showed that strict observance of dietary recommendations is associated with improved athletic performance [39,40,58]. However, most children and adolescents do not meet the recommendation of a healthy diet [59]. Only ca. 25% of children ate the recommended five meals per day [60]. It was observed that at the end of the study, the children ate fewer meals per day compared to the initial stage. Studies by Basiak-Rasała (2022) or Dolipska et al. (2018) similarly showed that older teenagers ate fewer meals per day than younger ones [61,62]. Breakfast is recognised as the most important meal of the day, especially in the case in school-aged children [63,64,65,66]. In this study, 63% of children declared eating breakfast every day, while ca. 9% did not eat breakfast at all. Breakfast skipping was also much more frequent in the control group than in the increased PA (10% vs. 6.67%). It was observed that children skipped breakfast more frequently with age, especially in the control group. Other studies reported a higher rate of children eating breakfast regularly [67,68]. The study of Noll et al. (2022) and others showed that 45.6% of young athletes skipped breakfast regularly [69]. Wądołowska et al. (2019) observed that over 17% of children aged 11–13 often skipped breakfast [70]. Unfortunately, the percentage of children regularly eating breakfast in Poland is decreasing continuously [67,71,72]. Mazur and Małkowska-Szkutnik (2018) as well as Myszkowska-Ryciak et al. (2019) indicated that the percentage of children eating breakfast every day was higher in 11-year-olds than in 13-year-olds (57.7%) [72,73]. This result is similar to obtained in this study. The study of Kim et al. (2016) found that eating breakfast, as well as fruit, vegetables and milk, was associated with improved education performance, while increased consumption of fizzy drinks, instant meals, fast foods and sweets were negatively correlated with school performance [74]. According to the Health Behaviour in School-aged Children (HBSC) 2017/2018 study, the lowest level of breakfast eating in school-aged children was observed in the Central Europe, while the highest in the Netherlands [75].

The results of this work showed that ca. 30% of children regularly ate sweets and savoury snacks between the main meals. Similar results were reported, inter alia, in the studies by Noll et al. (2020) and Coutinho et al., (2016) [69,76].

It is recommended to include at least three glasses of milk or a comparable amount of dairy products in the diet of school-aged children because of the high content of protein and calcium [60]. Nearly 40% of the studied children consumed milk and dairy products at least once a day, more frequently in the increased PA group than control. Interestingly, it was observed that the frequency of milk and dairy products consumption increased with age, especially in the increased PA group. Lower levels of dairy consumption were observed in the study by Basiak-Rasała et al., (2022), in which approximately 20% of children consumed milk and dairy products several times a day [61]. Stefanska et al. found that the recommended amounts of milk were consumed by only 30% of 11–12-year-olds and 20% of 13–15-year-olds [77]. The HELENA (Healthy Lifestyle in Europe by Nutrition in Adolescence) study showed that European adolescents consume less than two-thirds of the recommended amount of milk and dairy products [14].

The World Health Organization (WHO) recommends frequent eating of fruit and vegetables as an element of a healthy diet [78]. In this study, ca. 42% of children ate vegetables and fruit every day. Vegetables were more frequently ate by the increased PA children, and fruit–in control. In the study of Dortch et al. (2014), children participating in sports more frequently ate vegetables and fruit and were characterised by lower consumption of fizzy drinks than others [42]. The cited study pointed out a positive correlation between the healthy diet and PA level, which was also found in this study [42]. The results related to the frequency of vegetables and fruit eating obtained in this work conform to the results of Zadek et al. (2019) or Jonczyk et al. (2021) [79,80]. In the PRO GREENS study [81] involving 11-year-olds in 10 European countries, 23.5% of children met the recommendations for consumption of fruit and vegetables. In the same study, 53.3% of children did not consume vegetables on a daily basis [81]. Several large studies shown that consumption of fruit and vegetables was higher in children involved in sport activities, which was also observed in this study [82,83].

Nutrition of school-aged children should take into account the appropriate share of cereal products, especially wholemeal products, a valuable source of dietary fibre. A number of studies highlighted the particularly low level of wholemeal cereal products in the diet of children and adolescents [84,85,86]. Additionally, in this study, children more frequently consumed white than wholemeal bread and cereal products. At the same time, children with increased PA at school noticeably more rarely consumed white bakery products, and more frequently wholemeal products than the control ones.

Meat, poultry and fish are an important part of the diet and provide essential nutrients, such as proteins, iron, zinc, vitamin B12 and essential unsaturated fatty acids [87]. Processed meat products, such as ham, sausages were more frequently consumed by the control group children than increased PA. Children with increased PA at school ate poultry meat more often and red meat less often than the control group ones. Lower consumption of poultry meat was observed also in the study by Basiak-Rasała et al., (2022) [61].

Fish consumption is recommended at the level of 2 portions per week, which provide significant amount of omega-3 LC PUFA and improve learning performance [60,88]. An overall to little fish consumption was found. However, fish was more frequently eaten in the increased PA group than control. Nevertheless, it decreased over time. Even less frequent fish intake was reported in the study of Basiak-Rasała (2022) [61]. The studies by Całyniuk et al., (2014) and Dortch et al., (2014) showed that regular PA was associated with higher consumption of meat, fish, wholemeal bakery products and vegetables [41,42].

The frequency of fast food consumption increased with age, particularly in the control group. Fast foods were not consumed at all by 6% of children in the control group and 12% in the increased PA. Similar average results were obtained by Dolipska et al. (2018) [62]. Higher frequency of fast foods eating was observed in the study by Mendyk et al. (2018) [89].

Water is recommended as the basic drink for school-aged children [90,91]. Water was the most commonly selected drink in both groups. However, the control group children more drank sweetened, fizzy drinks than increased PA. Sweets and savoury snacks were one of the most frequently eaten snacks between meals. At the same time, increased PA children ate them less frequently than control ones. In the study by Mendyk et al. (2017) every day consumption of sweets was reported by 30% of adolescents [89].

More favourable eating behaviour was found to a greater extent in the increased PA group than control one. In the study of Roura et al. (2016) it was also observed more favourable eating behaviour in more physically active children than in less active ones [90]. The obtained results indicated that an increase in PA at school is associated with more favourable children’s eating behaviour and more active ways of spending leisure time.

It was observed that children in both GC and SC groups were inclined to spend more leisure time sedentary with age, especially GC ones. It is usual that children show screen-related sedentary behaviour. It is estimated than children spend in front of the screen approximately 4.5 h per day at aged 8–12, which increase to 6.5 h at age 13–18 spend per day [91].

Cvetković et al. (2021) similarly to other authors showed the trend to decrease quality of eating behaviour as well as leisure time PA level with age [92]. However, in this study such a trend was not observed in the increased PA at school children (SC). The obtained results confirmed hypothesis that additional compulsory PE lessons had a positive impact on eating behaviour and leisure time PA frequency in early adolescents during 2 years of observation. The increased organised PA at school is helpful in shaping a healthy lifestyle of early adolescents, which was shown to decrease the risk of obesity and hypertension [26,27]. To inhibit the growing trend of sedentary lifestyles and unhealthy diets among adolescents it is advisable to double the curricular number of PE lessons as it was shown in the present study.

Despite the significant trends shown, the study presented here has some limitations. The study included only 2 years of follow-up. It was planned to continue the study in the same group of children until finishing of primary school (age 15 years). However, the lockdown during the COVID-19 pandemic outbreak and remote learning caused the study to be discontinued. Furthermore, the study was limited to only a relatively small sample of children aged between 10 and 12 years. Another limitation is that not only the PA level, but also eating behaviour of families and the socio-economic status of families influence the eating behaviour, however, this study did not account for these factors. It was not asked the question whether the eating behaviour and the levels of physical activity are different during the summer holidays. Leisure time PA was assessed asking only frequency and type without measuring its duration and intensity.

## 5. Conclusions

More favourable eating behaviour was found in children with increased PA at school (SC) than in standard PA (GC). Children in the group of increased PA (SC) at school skip breakfast more rarely, more frequently ate vegetables and fruit, poultry meat and more rarely sweets, savoury snacks, fast food and fizzy drinks than standard ones (GC). Children rarely ate fish and approximately ^1^/_10_ of children in the group of increased PA (SC) and ¼ of standard PA (GC)did not eat fish at all. Water was the most common drink in both groups. During the course of the study the share of children with good and very good eating behaviour decreased in the standard PA group (GC) and increased in the increased PA (SC). The frequency of often undertaking PA (every day or most days a week) during leisure time decreased in the standard PA group (GC), while increased in the increased PA (SC). Increase in organized PA at school beneficially influenced early adolescents’ eating behaviour and frequency of undertaking leisure time PA during 2 years observation. It also increased early adolescents’ awareness of healthy diet. To inhibit the growing trend of sedentary lifestyles and unhealthy diet among adolescents it is advisable to double the curricular level of organized PA at school and strength nutritional education.

## Figures and Tables

**Table 1:** Eating behaviour of SC and GC children, average scores from the entire study period, % of particular ranks in the group.

No.	Questions	SC					GC					Statistical Measures *
		Ranks										
		1	2	3	4	5	1	2	3	4	5	
1.	Number of meals per day	0.00	0.00	14.33	52.00	33.66	0.00	0.00	18.67	50.67	30.66	χ^2^ = 1.592, df − 4; *p* = 0.810, VC = 0.127
2.	Eating of breakfast	6.67	7.67	9.00	13.33	63.33	10.00	7.00	10.00	11.00	62.00	χ^2^ = 12.220, df = 4; *p* = 0.020 VC = 0.072
3.	Eating of lunch at school	4.00	13.33	6.67	24.00	52.00	6.67	16.00	6.67	21.33	49.33	χ^2^ = 1.138, df = 4; *p* = 0.888, VC = 0.125
4.	Type of snacks eaten during the day	21.00	22.33	5.33	40.00	11.34	24.00	26.33	4.67	37.00	8.00	χ^2^ = 13.547, df − 4; *p* = 0.040, VC = 0.189
5.	Eating of milk and dairy products	2.33	6.00	17.00	36.00	38.67	3.33	1.67	21.00	38.66	35.33	χ^2^ = 8.243, df = 4; *p* = 0.043, VC = 0.289
6.	Eating of vegetables	0.00	0.00	12.33	41.67	46.00	7.66	0.66	17.00	36.02	38.66	χ^2^ = 10.042, df = 4; *p* = 0.039, VC = 0.319
7.	Eating of fruit	0.00	0.00	13.66	37.34	49.00	2.00	1.00	16.67	35.33	45.00	χ^2^ = 8.997, df = 4; *p* = 0.041, VC = 0.302
8.	Eating of white bread	0.67	1.00	12.67	60.33	25.33	2.33	1.33	6.33	64.68	25.33	χ^2^ = 9.535, df − 4; *p* = 0.038, VC = 0.160
9.	Eating of wholemeal bread	5.67	7.00	42.00	28.33	17.00	10.67	14.33	42.67	23.33	9.00	χ^2^ = 31.026, df − 4; *p* = 0.025, VC = 0.175
10.	Eating of coarse groats, wholemeal pasta, flakes, etc.	3.67	6.67	54.33	27.00	8.33	8.67	9.67	54.33	20.66	6.67	χ^2^ = 11.737, df − 4; *p* = 0.028, VC = 0.132
11.	Eating of fine groats, pasta, standard flakes	2.33	7.67	58.00	23.67	8.33	2.33	5.33	49.34	32.00	11.00	χ^2^ = 9.255, df − 2; *p* = 0.035, VC = 0.306
12.	Eating of eggs	6.33	9.67	55.00	23.67	5.33	6.33	5.00	56.33	23.67	8.67	χ^2^ = 1.667 df = 4; *p* = 0.794, VC = 0.130
13.	Eating of processed meat products	3.67	8.00	28.33	23.33	36.67	6.00	4.67	29.33	28.00	32.00	χ^2^ = 16.402, df = 4; *p* = 0.002, VC = 0.408
14.	Eating of poultry meat	0.00	6.67	29.33	20.00	44.00	1.66	9.67	32.67	14.00	42.00	χ^2^ = 12.570, df = 4; *p* = 0.036, VC = 0.126
15.	Eating of red meat	3.00	12.00	30.00	16.33	38.67	6.67	15.33	25.33	12.33	40.34	χ^2^ = 4.279, df − 4; *p* = 0.014 VC = 0.208
16.	Eating of fish	11.67	30.33	53.33	3.67	1.00	25.33	26.34	46.00	2.33	0.00	χ^2^ = 11.951, df − 4; *p* = 0.020, VC = 0.245
17.	Eating of fast foods	0.00	0.00	19.33	68.67	12.00	0.67	1.67	28.33	63.00	6.33	χ^2^ = 1.512, df = 4; *p* = 0.824, VC = 0.123
18.	Drinking beverages	6.33	17.00	17.00	4.67	55.00	11.67	15.33	10.00	6.33	56.67	χ^2^ = 13.547, df − 4; *p*= 0.047, VC = 0.189
Mean		4.30	8.63	26.53	30.22	30.32	7.55	8.97	26.41	28.90	28.17	χ^2^ = 10.952, df = 4; *p* = 0.016 VC = 0.098

Ranks: 5—very good, 4—good, 3—satisfactory, 2—bad, 1—very bad; GC—control group of standard PA at school (4 h a week); SC—increased PA at school (10 h a week); * Chi squared test (χ^2^); *p*-value ≤ 0.05; df—degrees of freedom; VC—V Cramera relationship strength.

**Table 2:** Eating behaviour of children in the initial assessment session (2017), % of particular ranks in the group.

No.	Questions	SC					GC					Statistical Measures *
		Ranks										
		1	2	3	4	5	1	2	3	4	5	
1.	Number of meals per day	0.00	0.00	21.00	38.00	41.00	0.00	0.00	17.00	46.00	37.00	χ^2^ = 1.338, df = 4; *p* = 0.846 VC = 0.116
2.	Eating of breakfast	2.00	5.00	6.00	18.00	69.00	3.00	5.00	7.00	14.00	71.00	χ^2^ = 1.805, df = 4; *p* = 0.937 VC = 0.182
3.	Eating of lunch at school	5.00	9.00	9.00	28.00	49.00	5.00	13.00	9.00	24.00	49.00	χ^2^ = 5.571, df = 4; *p* = 0.233 VC = 0.192
4.	Type of snacks eaten during the day	19.00	29.00	3.00	43.00	6.00	23.00	32.00	0.00	41.00	4.00	χ^2^ = 3.679, df = 4; *p* = 0.409 VC = 0.193
5.	Eating of milk and dairy products	4.00	13.00	20.00	32.00	31.00	4.00	0.00	26.00	39.00	31.00	χ^2^ = 14.472, df = 4; *p* = 0.005 VC = 0.383
6.	Eating of vegetables	0.00	0.00	9.00	51.00	40.00	6.00	0.00	24.00	39.00	31.00	χ^2^ = 15.559, df = 4; *p* = 0.003 VC = 0.397
7.	Eating of fruit	0.00	0.00	17.00	40.00	43.00	0.00	1.00	18.00	39.00	42.00	χ^2^ = 1.053, df = 4; *p* = 0.901 VC = 0.103
8.	Eating of white bread	0.00	0.00	12.00	71.00	17.00	0.00	0.00	3.00	71.00	26.00	χ^2^ = 7.283, df = 4; *p* = 0.121 VC = 0.271
9.	Eating of wholemeal bread	8.00	9.00	49.00	21.00	13.00	19.00	21.00	43.00	10.00	7.00	χ^2^ = 15.376, df = 4; *p* = 0.004 VC = 0.395
10.	Eating of coarse groats, wholemeal pasta, flakes, etc.	2.00	6.00	61.00	27.00	4.00	8.00	10.00	57.00	18.00	7.00	χ^2^ = 7.353, df= 4; *p* = 0.118 VC = 0.273
11.	Eating of fine groats, pasta, standard flakes	3.00	7.00	57.00	22.00	11.00	1.00	2.00	46.00	37.00	14.00	χ^2^ = 9.126, df = 4; *p* = 0.058 VC = 0.304
12.	Eating of eggs	6.00	11.00	61.00	22.00	0.00	0.00	2.00	66.00	30.00	2.00	χ^2^ = 15.658, df = 4; *p* = 0.003 VC = 0.398
13.	Eating of processed meat products	0.00	16.00	34.00	16.00	34.00	0.00	12.00	30.00	28.00	30.00	χ^2^ = 4.344, df = 4; *p* = 0.361 VC = 0.210
14.	Eating of poultry meat	0.00	12.00	31.00	15.00	42.00	0.00	14.00	33.00	11.00	42.00	χ^2^ = 1.740 df = 4; *p* = 0.783 VC = 0.132
15.	Eating of red meat	5.00	16.00	24.00	15.00	40.00	5.00	16.00	23.00	14.00	42.00	χ^2^ = 2.016, df = 4; *p* = 0.732 VC = 0.143
16.	Eating of fish	9.00	18.00	64.00	6.00	3.00	26.00	18.00	53.00	3.00	0.00	χ^2^ = 13.291, df = 4; *p* = 0.009 VC = 0.367
17.	Eating of fast foods	0.00	0.00	22.00	68.00	10.00	0.00	0.00	26.00	70.00	4.00	χ^2^ = 2.933, df = 4; *p* = 0.569 VC = 0.172
18.	Drinking beverages	9.00	18.00	14.00	5.00	54.00	16.00	23.00	10.00	7.00	44.00	χ^2^ = 4.590, df = 4; *p* = 0.331 VC = 0.215
Mean		4.00	9.39	28.55	29.89	28.17	6.44	9.39	27.28	30.06	26.83	χ^2^ = 0.871, df = 4; *p* = 0.927 VC = 0.194

Ranks: 5—very good, 4—good, 3—satisfactory, 2—bad, 1—very bad; GC—control group of standard PA at school (4 h a week); SC—increased PA at school (10 h a week); * Chi squared test (χ^2^); *p*-value ≤ 0.05; df–degrees of freedom; VC—V Cramera relationship strength.

**Table 3:** Eating behaviour of SC and GC children in the middle assessment session (2018), % of particular ranks in the group.

No.	Questions	SC					GC					Statistical Measures *
		Ranks										
		1	2	3	4	5	1	2	3	4	5	
1.	Number of meals per day	0.00	0.00	12.00	53.00	35.00	0.00	0.00	18.00	53.00	29.00	χ^2^ = 11.762, df = 4; *p* = 0.009 VC = 0.133
2.	Eating of breakfast	8.00	10.00	8.00	12.00	62.00	13.00	6.00	11.00	10.00	60.00	χ^2^ = 5.874, df = 4; *p* = 0.048 VC = 0.021
3.	Eating of lunch at school	4.00	15.00	7.00	22.00	5200	7.00	17.00	6.00	21.00	49.00	χ^2^ = 1.132, df = 4; *p* = 0.029 VC = 0.182
4.	Type of snacks eaten during the day	21.00	19.00	5.00	42.00	13.00	23.00	24.00	6.00	37.00	10.00	χ^2^ = 1.471, df = 4; *p* = 0.831 VC = 0.122
5.	Eating of milk and dairy products	0.00	3.00	12.00	40.00	45.00	4.00	0.00	13.00	43.00	40.00	χ^2^ = 7.442, df = 4; *p* = 0.114 VC = 0.274
6.	Eating of vegetables	0.00	0.00	11.00	42.00	47.00	4.00	0.00	11.00	41.00	44.00	χ^2^ = 10.110, df = 4; *p* = 0.031 VC = 0.024
7.	Eating of fruit	0.00	0.00	6.00	37.00	57.00	1.00	0.00	12.00	36.00	51.00	χ^2^ = 3.347, df = 4; *p* = 0.501 VC = 0.184
8.	Eating of white bread	0.00	0.00	12.00	56.00	32.00	2.00	2.00	6.00	64.00	26.00	χ^2^ = 17.154, df = 4; *p* = 0.028 VC = 0.269
9.	Eating of wholemeal bread	5.00	6.00	42.00	27.00	20.00	7.00	9.00	49.00	24.00	11.00	χ 2 = 4.261, df = 4; *p* = 0.371 VC = 0.207
10.	Eating of coarse groats, wholemeal pasta, flakes, etc.	2.00	4.00	55.00	29.00	10.00	4.00	7.00	59.00	26.00	4.00	χ^2^ = 4.360, df = 4; *p* = 0.359 VC = 0.210
11.	Eating of fine groats, pasta, standard flakes	2.00	4.00	57.00	27.00	10.00	1.00	4.00	45.00	38.00	12.00	χ^2^ = 3.788, df = 4; *p* = 0.453 VC = 0.195
12.	Eating of eggs	0.00	0.00	59.00	26.00	15.00	0.00	0.00	61.00	20.00	19.00	χ^2^ = 1.286, df = 4; *p* = 0.863 VC = 0.114
13.	Eating of processed meat products	0.00	0.00	30.00	26.00	44.00	0.00	0.00	34.00	30.00	36.00	χ^2^ = 1.335, df = 4; *p* = 0.855 VC = 0.116
14.	Eating of poultry meat	0.00	2.00	29.00	25.00	44.00	0.00	5.00	35.00	17.00	43.00	χ^2^ = 13.585, df = 4; *p* = 0.035 VC = 0.190
15.	Eating of red meat	0.00	11.00	34.00	16.00	39.00	7.00	15.00	25.00	13.00	40.00	χ^2^ = 6.896, df = 4; *p* = 0.141 VC = 0.264
16.	Eating of fish	17.00	38.00	45.00	0.00	0.00	21.00	33.00	46.00	0.00	0.00	χ^2^ = 0.784, df = 4; *p* = 0.940 VC = 0.007
17.	Eating of fast foods	0.00	0.00	20.00	66.00	14.00	0.00	0.00	27.00	66.00	7.00	χ^2^ = 3.375 df = 4; *p* = 0.490 VC = 0.185
18.	Drinking beverages	6.00	24.00	16.00	6.00	48.00	10.00	16.00	9.00	10.00	55.00	χ^2^ = 9.035, df = 4; *p* = 0.006 VC = 0.247
Mean		3.60	7.56	25.56	30.67	32.61	5.78	7.67	26.27	30.50	29.78	χ^2^ = 10.468, df = 4; *p* = 0.023 VC = 0.068

Ranks: 5—very good, 4—good, 3—satisfactory, 2—bad, 1—very bad; GC—control group of standard PA at school (4 h a week); SC—increased PA at school (10 h a week); * Chi squared test (χ^2^); *p*-value ≤ 0.05; df—degrees of freedom; VC—V Cramera relationship strength.

**Table 4:** Eating behaviour of children in the final assessment session (2019), % of particular ranks in the group.

No.	Questions	SC					GC					Statistical Measures *
		Ranks										
		1	2	3	4	5	1	2	3	4	5	
1.	Number of meals per day	0.00	0.00	10.00	65.00	25.00	0.00	0.00	21.00	53.00	26.00	χ^2^ = 9.143, df = 4; *p* = 0.020 VC = 0.228
2.	Eating of breakfast	10.00	8.00	13.00	10.00	59.00	10.00	7.00	10.00	11.00	62.00	χ^2^ = 13.215, df = 4; *p* = 0.010 VC= 0.386
3.	Eating of lunch at school	3.00	16.00	4.00	22.00	55.00	8.00	18.00	5.00	19.00	50.00	χ^2^ = 11.425, df = 4; *p* = 0.042 VC = 0.002
4.	Type of snacks eaten during the day	23.00	19.00	8.00	35.00	15.00	26.00	23.00	8.00	33.00	10.00	χ^2^ = 1.623, df = 4; *p* = 0.804 VC = 0.128
5.	Eating of milk and dairy products	3.00	2.00	19.00	36.00	40.00	2.00	5.00	24.00	34.00	35.00	χ^2^ = 12.457, df = 4; *p* = 0.052 VC = 0.150
6.	Eating of vegetables	0.00	0.00	17.00	32.00	51.00	13.00	2.00	16.00	29.00	41.00	χ^2^ = 16.260, df = 4; *p* = 0.002 VC = 0.406
7.	Eating of fruit	0.00	0.00	18.00	35.00	47.00	5.00	2.00	20.00	31.00	42.00	χ^2^ = 12.628, df = 4; *p* = 0.006 VC = 0.278
8.	Eating of white bread	2.00	3.00	14.00	54.00	27.00	5.00	2.00	10.00	59.00	24.00	χ^2^ = 2.550, df = 4; *p* = 0.635 VC = 0.160
9.	Eating of wholemeal bread	4.00	6.00	35.00	37.00	18.00	6.00	13.00	36.00	36.00	9.00	χ^2^ = 9.006, df = 4; *p* = 0.028 VC = 0.246
10.	Eating of coarse groats, wholemeal pasta, flakes, etc.	7.00	10.00	47.00	25.00	11.00	14.00	12.00	47.00	18.00	9.00	χ^2^ = 3.854, df = 4; *p* = 0.426 VC = 0.197
11.	Eating of fine groats, pasta, standard flakes	2.00	12.00	60.00	22.00	4.00	5.00	10.00	57.00	21.00	7.00	χ^2^ = 2.385, df = 4; *p* = 0.665 VC = 0.155
12.	Eating of eggs	13.00	18.00	45.00	23.00	1.00	19.00	13.00	42.00	21.00	5.00	χ^2^ = 4.792, df = 4; *p* = 0.309 VC = 0.220
13.	Eating of processed meat products	11.00	8.00	21.00	28.00	32.00	18.00	2.00	24.00	26.00	30.00	χ^2^ = 5.268, df = 4; *p* = 0.228 VC = 0.231
14.	Eating of poultry meat	0.00	6.00	28.00	20.00	46.00	5.00	10.00	30.00	14.00	41.00	χ^2^ = 8.303, df = 4; *p* = 0.021 VC = 0.290
15.	Eating of red meat	4.00	9.00	32.00	18.00	37.00	8.00	15.00	28.00	10.00	39.00	χ^2^ = 3.136, df = 4; *p* = 0.535 VC = 0.178
16.	Eating of fish	9.00	35.00	51.00	5.00	0.00	29.00	28.00	39.00	4.00	0.00	χ^2^ = 13.015, df = 4; *p* = 0.011 VC = 0.363
17.	Eating of fast foods	0.00	0.00	16.00	72.00	12.00	2.00	5.00	32.00	53.00	8.00	χ^2^ = 16.021, df = 4; *p* = 0.003 VC = 0.403
18.	Drinking beverages	4.00	9.00	21.00	3.00	63.00	9.00	7.00	11.00	2.00	71.00	χ^2^ = 9.975, df = 4; *p* = 0.038 VC = 0.246
Mean		5.27	8.94	25.50	30.11	30.18	10.23	9.67	25.56	26.33	28.21	χ^2^ = 12.772, df = 4; *p* = 0.055 VC = 0.134

Ranks: 5—very good, 4—good, 3—satisfactory, 2—bad, 1—very bad; GC—control group of standard PA at school (4 h a week); SC—increased PA at school (10 h a week); * Chi squared test (χ^2^); *p*-value ≤ 0.05; df—degrees of freedom; VC—V Cramera relationship strength.

**Table 5:** Frequency of leisure time physical activity, % of particular ranks in the group.

Average Age (Years)	SC					GC					Statistical Measures *
	Ranks										
	1	2	3	4	5	1	2	3	4	5	
10.27	0.00	0.00	33.00	35.00	32.00	4.00	5.00	10.27	30.00	28.00	χ^2^ = 9.651; df = 4; *p* = 0.040 VC = 0.258
11.27	0.00	0.00	12.00	49.00	39.00	2.00	2.00	11.27	38.00	25.00	χ^2^ = 18.253; df = 4; *p* = 0.011 VC = 0.143
12.27	0.00	7.00	19.00	41.00	33.00	8.00	5.00	12.27	30.00	22.00	χ^2^ = 16.978; df = 4; *p* = 0.002 VC = 0.138
Mean	0.00	2.33	21.33	41.67	34.67	4.66	4.00	33.67	32.67	25.00	χ^2^ = 2.612; df − 4; *p* = 0.013; VC = 0.128

Ranks: 5—very good, 4—good, 3—satisfactory, 2—bad, 1—very bad; GC—control group of standard PA at school (4 h a week); SC—increased PA at school (10 h a week); * Chi squared test (χ^2^); *p*-value ≤ 0.05; df—degrees of freedom; VC—V Cramera relationship strength.

## Data Availability

Data is available upon request.
